# Diagnosis and Treatment of a Symptomatic Posterior Cruciate Ganglion Cyst in a Child with Autism

**DOI:** 10.1155/2019/9192347

**Published:** 2019-03-05

**Authors:** Valerio Andreozzi, Edoardo Monaco, Fabio Conteduca, Raffaele Iorio, Daniele Mazza, Piergiorgio Drogo, Andrea Ferretti

**Affiliations:** Department of Orthopaedics, Sapienza University of Rome, Italy

## Abstract

**Introduction:**

Intra-articular ganglion cysts of the knee joint are rare and mostly incidental findings in magnetic resonance imaging (MRI) or arthroscopy. Posterior cruciate ligament (PCL) ganglion cyst in a child is an extremely rare finding, and to the best of our knowledge, only one case has been described in the literature. We report a case of a large intra-articular ganglion cyst of the knee arising from the PCL in an autistic child.

**Case Presentation:**

An 8-year-old Caucasian boy affected by autism presented with nontraumatic knee pain. His parents, observing child's gait, reported recurrent limp while walking, sometimes accompanied by knee locking. Clinical examination was hindered by the noncompliance of the patient and revealed painful limitation of terminal flexion and extension. MRI scans showed a large ganglion cyst located in the intercondylar notch. Arthroscopy confirmed an intrasubstance PCL ganglion cyst, extending both anteriorly and posteriorly. Complete excision of the cyst was performed, with full recovery of the child and no recurrence.

**Conclusion:**

In pediatric patients with pain or limited knee range of motion, physicians should consider the possibility of a ganglion cyst arising from the PCL, despite its rarity. Arthroscopic excision is a safe and effective procedure that guarantees a complete recovery of the patient with the lowest rate of recurrence.

## 1. Introduction

The majority (78–90%) of ganglions in adults are located in the wrist, but they can occur almost anywhere in the musculoskeletal system, including the knee [1].

Ganglion cysts (GC) arising from the cruciate ligaments are rare, with a reported prevalence of 0.36% when detected by MRI and 0.8% by knee arthroscopy [2]. Posterior cruciate ligament (PCL) ganglion cysts occur less frequently than those arising from the anterior cruciate ligament (ACL) [3, 4]. Krudwig et al., in a study conducted on over 8000 knee arthroscopies in a 15-year period, reported a prevalence of ganglion cysts originating from the ACL more than three times higher than the PCL ones [3].

Those lesions are mainly diagnosed in patients aged between 20 and 40 years [3, 5, 6], and a male preponderance has been reported [5].

To the best of our knowledge, only one case of pediatric ganglion cyst of the PCL has been reported in the literature [7].

In this report, we present an 8-year-old boy with autism spectrum disorder (ASD) and a symptomatic left knee, who was found to have a large ganglion cyst of the PCL. We discuss the clinical presentation, imaging findings, histopathology, surgical management, and postoperative rehabilitation.

## 2. Case Report

**Figure 1:**
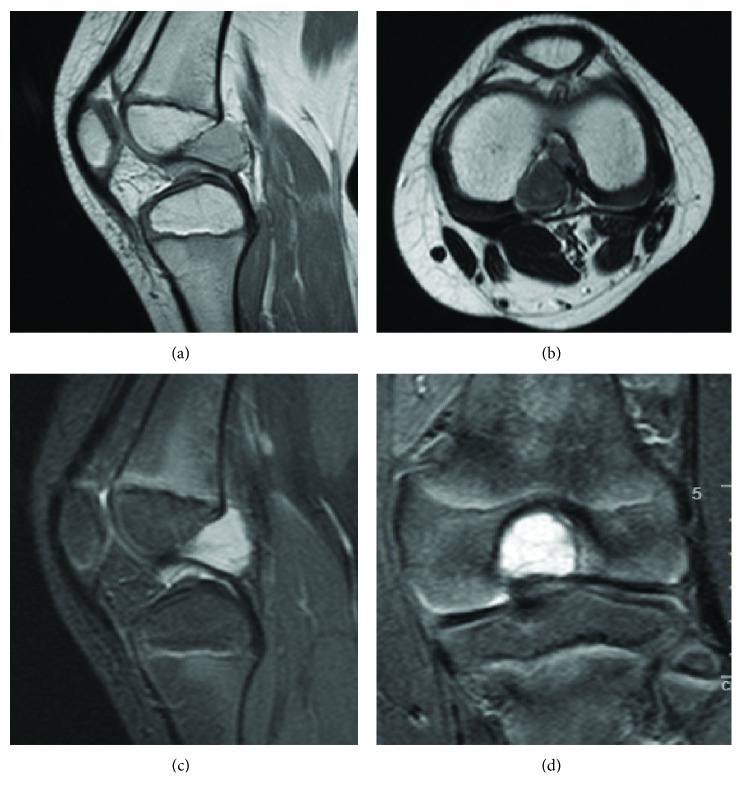
Preoperative magnetic resonance images of the left knee revealed large round-shaped cystic mass encasing the PCL with homogeneous low-signal intensity on sagittal proton density-weighted image (PDWI) (a) and on turbo spin echo (TSE) imaging (b). The lesion appears highly hyperintense on sagittal and coronal turbo inversion recovery magnitude (TIRM) images (c, d).

An 8-year-old Caucasian boy with ASD presented to our clinic with a history of limping and recurrent left knee pain mainly in the back of the knee, exacerbated with activity and partially alleviated with rest. Clinical examination was difficult to perform, due to the strong opposition of the autistic child. His left knee was not swollen and no joint line tenderness was elicited. Range of motion (ROM) of the left knee was slightly limited in extension compared with the opposite side, and hyperflexion was painful and slightly limited as well. The McMurray, Lachman, and varus/valgus stress tests were all negative. Plain radiographs were performed and resulted normal. A second-level imaging was needed, but the presence of ASD complicated the execution of the test, so MRI of the left knee was performed under general anesthesia with sevoflurane. Scans revealed a 29 mm × 16 mm × 17 mm well-defined septated cyst located in the intercondylar notch between the ACL and PCL, abutting predominantly posteriorly to the PCL. The round-shaped cystic mass encasing the PCL depicted homogeneous low-signal intensity, slightly hyperintense relative to the muscles, on proton density-weighted image (PDWI) and on turbo spin echo (TSE) imaging and high-signal intensity on turbo inversion recovery magnitude (TIRM) images (Figure 1).

**Figure 2:**
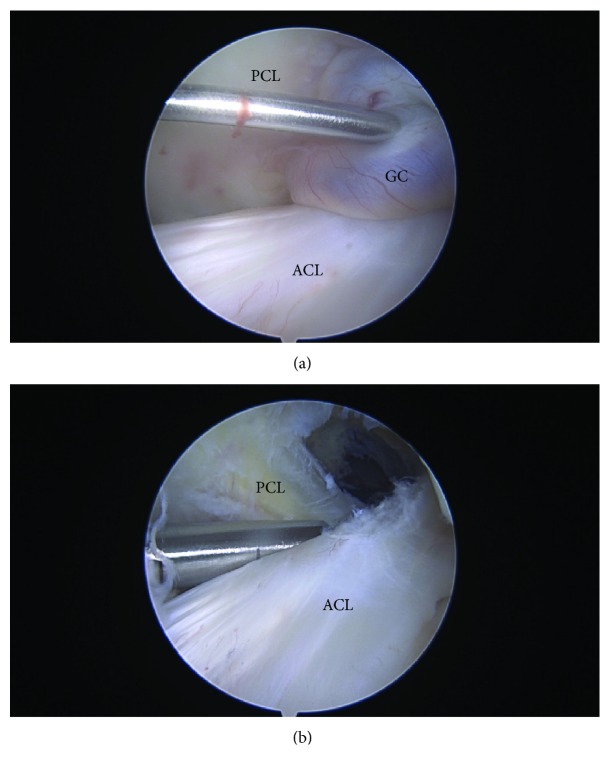
A round-shaped ganglion cyst (GC) as seen on arthroscopy from the anterolateral portal, before (a) and after (b) excision. The GC was located between the anterior cruciate ligament (ACL) and the posterior cruciate ligament (PCL), tightly attached to the PCL.

Arthroscopic surgery was performed under general anesthesia and a tourniquet was used. The location of the cyst correlated with the MRI findings. Arthroscopic examination, performed through standard anterolateral and anteromedial portals, revealed a large white encapsulated ganglion cyst, with blood vessels on the surface, filling the femoral notch (Figure 2). The cystic mass, arising from the PCL, enveloped PCL fibers and extended posteriorly in intimate connection with the posterior capsule. Both cruciate ligaments appeared intact as medial and lateral menisci were without tears under arthroscopic examination. Before excising the cyst, we used punch forceps to obtain a specimen for histopathology, then a motorized shaver was used to excise the cyst completely from the PCL and the posterior capsule. After debridement, any persistent bleeding has been controlled using a radiofrequency ablation probe to ensure hemostasis. A pressure dressing was applied onto the affected knee after the operation.

**Figure 3:**
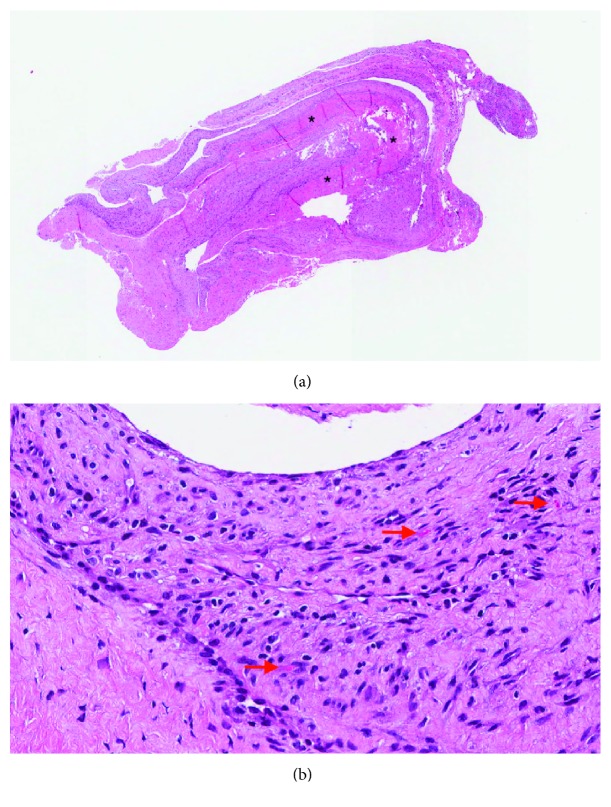
Hematoxylin-eosin staining shows that the cyst wall is lined by synovial cells and is composed of dense fibroconnective tissue, widespread thick bundles of collagen (^∗^), and capillary proliferation (red arrow). (a) ×10 and (b) ×40.

Histologic examination revealed the proliferation of synovial cells lined with dense fibroconnective tissue, widespread thick bundles of collagen and capillary proliferation, confirming the diagnosis of posterior cruciate ligament ganglion cyst (Figure 3). The symptoms improved immediately after the operation, and the patient was addressed to a short postoperative rehabilitation program due to his neurological condition. At 3 months postoperatively, the patient had an International Knee Documentation Committee (IKDC) score of 97, as he was able to perform all activities of daily living, including squatting and sitting in the crossed leg position, and had full ROM. Twelve months after successful arthroscopic excision, no recurrence was detected under clinical examination.

## 3. Discussion

Intra-articular synovial cysts of the knee joint are uncommon and mostly incidental findings during arthroscopy or MRI [5, 8, 9]. In particular, ganglion cysts in the knee of pediatric patients, as encountered in the present case, are very rare.

In 1924, Caan described for the first time an asymptomatic ganglion of the ACL during a knee autopsy of a man [10]. Favorito and Schwend reported one of the youngest patient in the literature to be diagnosed with an intercruciate ligament ganglion cyst in 2001 [11], but the youngest patient in the medical literature was a child of two years with a massive intra-articular knee cyst and an aberrant ACL origin [12]. Both children had a past behavior of refusal to bear full weight and limping, but families denied any history of trauma.

The exact pathogenesis of gangliar cyst is still debated. There are theories suggesting mucinous degeneration of connective tissue, synovial herniation, and congenitally displaced synovial tissue [9, 13, 14], but one of the most accredited is that joint capsules or ligaments produce mucin as a reaction to stress, stimulating the production of modified synovial cells, fibroblasts, or mesenchymal cells [1, 15]. In light of this, repeated minor knee trauma seems to play an important role [3, 5, 9, 15].

Thus, the predominance of ganglion cysts in adult males indicates that trauma might be a major determinant in the pathogenesis; females instead are historically considered to be less likely to suffer trauma and sporting injuries, as shown in a meta-analysis by Tolin and Foa [16]. Probably, in our case, as in the majority of pediatric intra-articular synovial cysts, the origin of the ganglion cyst itself remains enigmatic. Factors supporting the traumatic origin of the cyst in our patient come from the observations that children with ASD show deficient sensory perception relative to internal or external stimuli [17] and highly individualized asymmetrical lower extremity angular joint positions during gait [18], which may induce tripping or falling and be a cause of repeated trauma over time. This hypothesis, though, could not be confirmed in our case, since no trauma was reported by the family, and the congenital theory might be also considered.

Ganglion cysts of the knee joint are difficult to diagnose due to the lack of specific symptoms and signs. When symptomatic, they present with nonspecific symptoms such as knee pain, locking, limitation of knee range of motion, or joint-line tenderness, simulating other intra-articular pathology such as meniscal or chondral lesions [19]. Differential diagnoses include pigmented villonodular synovitis, meniscal tears and cysts, synovial haemangioma, synovial sarcoma, cruciate mucoid degeneration and synovial chondromatosis; hence, biopsy for histology is advisable [3]. Cysts located mainly anterior to cruciate ligaments tend to limit extension of the knee, whereas those located predominately posterior to the cruciate ligaments tend to limit knee flexion. Our patient suffered from limping and recurrent pain in the back of the knee, and knee motion was slightly limited both in extension and in flexion, probably due to the large size of the cyst, which predominantly abutted posteriorly but also impinged anteriorly against the intercondylar roof. Intra-articular synovial cysts can also be asymptomatic or present with nonspecific knee pain like the 4-year-old boy described by Bui-Mansfield and Youngberg [8], who reported one of the first cases with a clear diagnose of PCL ganglion cyst in a child; the boy suffered from a dull and aching pain localized at the back of his knee, with a full ROM and a limp while walking. We could hypothesize that due to the small size of the cyst he did not experience any limitation of ROM.

Arthroscopic resection, debridement, or excision is the preferred and most successful procedure, with a very low rate of recurrence [3, 20, 21]. To date, recurrence appears to be unusual following arthroscopic resection; Mao et al. found no recurrence in their case series for ganglion cyst of the cruciate ligaments (31 patients) [5], and no recurrence was observed in the 85 cases of Krudwig et al. [3].

To ensure a full debridement of the cyst, we used a shaver and a radiofrequency ablation probe, which also reduced the risk of acute hematoma [5].

Delay in surgery can make the procedure more technically demanding and may require excision extending into the cruciate ligaments, eventually leading to ACL or PCL rupture [22].

Preoperative MRI aids the surgical approach planning in regard to the need for special instruments, which in this case was a radiofrequency ablator, in order to avoid bleeding from synovial blood vessels; sometimes the use of a posterior transseptal portal is a valid option for the excision of lesions located posteriorly to the PCL [23].

Nonoperative methods of treating these lesions are computed tomography (CT) or ultrasound-guided percutaneous aspiration [24]. The main advantages of those nonoperative procedures include quicker recovery time and decreased invasiveness [9]. However, the risk of recurrence is higher than arthroscopy [25], and it may have been technically demanding in our case as the majority of the cyst was located posteriorly and part of it was interspersed in the ACL fibers. Regarding the rehabilitation after the procedure, our patient presented some difficulties in the recovery; since children with ASD present, as major symptom of their disease, impairment in walking, fine and gross motor skills, and postural control [26], they are often excessively perceptive to touch and may have very low tolerance to pain [27].

## 4. Conclusion

In conclusion, ganglion cysts of the PCL are very rare especially in the pediatric population. When active, pediatric patients present with recurrent or persistent limp, pain, and eventually limited ROM, the aforementioned disease should be considered in the initial differential diagnosis and an MRI scan of the affected knee is recommended. Arthroscopic excision is a safe and effective option even in children with PCL ganglia, allowing a complete recovery, as well as in patients with autism spectrum disorder.
